# Dieting and Disinhibited Eating Patterns in Adult Women with Normal Body Weight: Does Rumination Matter?

**DOI:** 10.3390/nu13072475

**Published:** 2021-07-20

**Authors:** Justyna Waliłko, Paulina Bronowicka, Jinbo He, Anna Brytek-Matera

**Affiliations:** 1 Institute of Psychology, University of Wroclaw, 50-527 Wroclaw, Poland; 291938@uwr.edu.pl (J.W.); 290259@uwr.edu.pl (P.B.); 2 School of Humanities and Social Science, The Chinese University of Hong Kong, Shenzhen 518172, China; hejinbo@cuhk.edu.cn; 3 Faculty of Psychology in Katowice, SWPS University of Social Sciences and Humanities, 40-326 Katowice, Poland

**Keywords:** dieting, emotional eating, uncontrolled eating, rumination, weight, restraint theory

## Abstract

Dieting and disinhibited eating patterns are presented in both clinical and nonclinical samples. Repetitive negative thinking (i.e., rumination) may lead to maladaptive eating behaviors. While numerous studies have focused on dieting and disinhibited eating behaviors in clinical samples, less is known about these behaviors in nonclinical samples with normal body weight. Therefore, the present study aimed to explore how dieting, uncontrolled eating and emotional eating are related to rumination in adult women with normal body weight. One hundred eighty-eight women (*M*_age_ = 29.46 ± 8.94; *M*_BMI_ = 23.16 ± 4.04) were involved in the current study. The Eating Attitudes Test, the Three-Factor Eating Questionnaire-R18 and the Perseverative Thinking Questionnaire were administered to the participants. The results showed that repetitive negative thinking was a partial mediator in the relationship between dieting and uncontrolled eating, as well as in the relationship between dieting and emotional eating. Targeting repetitive negative thinking may be important for reducing disinhibited eating patterns in women with normal body weight.

## 1. Introduction

Dieting is defined as the intentional effort to control caloric intake by avoiding food consumption in order to maintain or lose weight [1]. There are three main categories of dietary strategies to promote weight loss: (1) diets based on the manipulation of macronutrient content (i.e., low-fat diet, low-carbohydrate diet); (2) diets based on the restriction of specific foods or food groups (i.e., gluten-free diet, plant-based diet); and (3) diets based on the manipulation of timing (i.e., fasting) [2]. Many women, especially adolescent girls and young women, diet because of body dissatisfaction and the desire to change both body size and shape [3].

### 1.1. Disinhibited Eating Patterns

Theoretical and empirical evidence suggests that dietary restraint may lead to an excessive intake of food [4,5,6], disinhibited eating and overconsumption [7]. Disinhibition is the tendency to overeat in response to different stimuli, and can occur in a variety of circumstances (e.g., exposure to palatable food cues, emotional distress) [8]. Dietary disinhibition was found to be associated with a loss of control, eating in response to emotional distress and overeating [7]. Uncontrolled eating (sometimes called external eating) is characterized by overconsumption, with the feeling of loss of control, of the amount of food intake products in response to external food cues (the sight and smell of attractive food) instead of internal cues such as the level of hunger and satiety [9], while emotional eating means the tendency to overeat in response to negative emotions (e.g., anxiety) [10].

Uncontrolled eating and emotional eating can be conceptualized as a form of disinhibition, where individuals feel compelled to eat in response to external or internal (emotional) cues and lack control over inhibiting this behavior [11]. Both uncontrolled eating and emotional eating are behaviors mostly exhibited by women, including normal-weight, overweight, and underweight individuals [12,13]. Cross-sectional studies [14] and longitudinal data [15] have found that in women with normal body weight, the interaction between restrained and disinhibited eating (uncontrolled eating, emotional eating) predicts food intake, with dietary restraint moderating the impact of uncontrolled eating.

### 1.2. Repetitive Negative Thinking (Rumination)

The control of food intake and the preoccupation with eating may cause intrusive food-related thoughts. Repetitive negative thinking, commonly called rumination, is a cognitive process involving repetitive thoughts about one’s problems or negative experiences and is characterized by repetitiveness, intrusiveness and difficulty in disengaging from this negative thinking [16]. Rumination has been implicated in mood and anxiety disorders [17,18], and there is a growing body of research on rumination in relation to eating disorder pathology [19,20,21]. Disorder-specific thoughts, focusing particularly on the control of eating, weight and shape [22,23], are presented in women with eating disorders and are likely to contribute to the increased severity in eating disorder symptomatology [24]. There are surprisingly few studies examining the process of rumination in normal-weight individuals.

Previous research has found effects of environmental food cues and food-related thoughts on eating behavior in healthy young women as well as women with eating disorders and overweight individuals [25,26]. Normal-weight individuals exhibit decreased susceptibility to food cue exposure, relative to overweight individuals [26]. They reported decreased desire for high-calorie food after exposure to tempting food words, whereas overweight individuals reported increased craving. In addition, normal-weight individuals respond to food-related cues with cognitions associated with eating less after a “thought–shape fusion” induction (imagining eating high-calorie food leads individuals to feel fatter, and to perceive weight gain and moral wrongdoing). In contrast, overweight individuals appear to respond to food-related cues with increased food desire with the absence of cognitions that may motivate reduced food consumption of high-calorie foods [26].

### 1.3. Objective of the Current Study

The evidence of negative repetitive thinking in the context of disinhibited eating patterns in normal-weight individuals is limited. Therefore, the present study aimed to examine the mediator effect of rumination on the relationship between dieting and both uncontrolled eating and emotional eating in a community sample of adult women.

Based on restraint theory [4] and taking into consideration that repetitive negative thinking is associated with disordered eating behaviors [21,27] and disinhibited eating patterns in normal-weight samples [28], the following hypotheses were proposed:

H1: A high level of concern about dieting is associated with more frequent uncontrolled eating and emotional eating, and higher levels of rumination.

H2: The relationship between a high level of concern about dieting and more frequent disinhibited eating patterns is mediated by higher levels of negative repetitive thinking among women with the body mass index (BMI) range from 18.5 to 24.99 kg/m^2^ (normal weight) [29].

Dietary restraint, which assesses both concern with dieting and weight fluctuations, could in part explain failures in maintaining normalized eating [26]. Thus, a more detailed knowledge of variables related to dieting may point out targets for improved eating interventions to increase normalized eating behavior in normal-weight individuals.

## 2. Materials

### 2.1. Participants

Participants were 21- to 55-year-old normal-weight women (*M*_age_ = 29.46 ± 8.94; *M*_BMI_ = 23.16 ± 4.04). One hundred eighty-eight community adult women participated in the present study (response rate = 83%). The purposive sampling method was used in the present study. Female participants met the following inclusion/exclusion criteria: (1) age between 21 and 55 years; (2) non-vegetarian and tries to eat healthily on a regular basis, but also enjoys eating junk food and snacks; (3) not allergic to major groups of food (e.g., gluten allergy); (4) no current or past-year history of major depression (which can affect appetite and weight) or one of three major eating disorders (anorexia, bulimia, binge eating disorder); (5) no abuse of drugs or alcohol (which can affect appetite and weight); (6) and no lifetime history of psychosis, mania, hypomania, bipolar disorders, or suicidality, defined using the Mini International Neuropsychiatric Interview (MINI) [30]. The electronic version of the MINI was used through the Nview Health portal (portal.nviewhealth.com). The participants, with an identifying number, filled out the answers themselves. Detailed instructions on the online interview process were given. A PDF of generated participant data was automatically sent to the interviewer’s email address (A.B.-M.). Only individuals with a low total score on the interview sections were invited to participate in the present study. The screening inclusion/exclusion criteria were made at the baseline of the study. The baseline study was conducted via an online platform (SurveyMonkey^®^, San Mateo, CA, USA) and consisted of three elements: first, confirming eligibility against inclusion and exclusion criteria; second, consent; and third, data collection.

The current study is part of the Harmonia 10 research project funded by the National Science Centre (Poland; grant no. 2018/30/M/HS6/00022), which focuses on the impact of negative affect on eating behavior in ecological and natural sittings. The first phase of the Harmonia 10 research project contained several self-administered questionnaires completed in a natural setting and assessed demographic variables and individual differences (eating behaviors, disordered eating behaviors, anxiety, negative affect, affect regulation, rumination).

### 2.2. Procedure

Collecting research data through traditional paper-and-pencil methods was not possible during the COVID-19 pandemic, so an online survey was used in the present study (SurveyMonkey^®^). Only community members who met study eligibility criteria participated in the present study. The participants were recruited through university centers, health clubs, fitness gyms (using flyers) and through social media such as Facebook. Participants received notice about the research with an announcement including all necessary information about the study. They received an email with the online link to the study. They were invited to visit a website that directed them to the consent form, information form and questionnaires. All participants offered their informed consent before starting the survey (by ticking a respective box at the first page of the online survey) and responded voluntarily to the survey. Women were informed about the anonymity of the study, and about the possibility of resignation at any stage of the study.

The research protocol of the Harmonia 10 research project was designed and conducted in accordance with the guidelines of the Declaration of Helsinki and was approved by the Research Ethics Committee at the Institute of Psychology, University of Wroclaw, Poland (decision number IPE 0019).

### 2.3. Methods

#### 2.3.1. The Eating Attitudes Test

The Eating Attitudes Test (EAT-26) [1,31] is one of the most widely used screening instruments to assess symptoms of eating disorders. It has also been used in nonclinical samples to detect characteristics and concerns related to eating disorders [32]. The EAT-26 contains 26 items in three subscales: (1) dieting (the pathological avoidance of fattening foods and body shape preoccupation); (2) bulimia and food preoccupation (bulimic behaviors and thoughts about food); and (3) oral control (pertains to self-control regarding eating and perceived pressure from others to gain weight) [1]. A total score of 20 or more indicates a risk of eating disorders (e.g., anorexia nervosa, bulimia nervosa and binge eating disorder). We used the Polish version of the EAT-26 [31] (α_total score of the EAT-26_ = 0.80). In the present study, internal reliabilities for the three subscales were as follows: dieting Cronbach’s α = 0.811, bulimia and food preoccupation Cronbach’s α = 0.693 and oral control Cronbach’s α = 0.520. The total EAT-26 Cronbach’s alpha was 0.834. In the present study, we only used one subscale from the EAT-26, dieting, for assessing dietary restriction.

#### 2.3.2. The Three-Factor Eating Questionnaire-R18

The Three-Factor Eating Questionnaire-R18 (TFEQ-R18) [33,34] is one of the most widely used measures to assess eating behaviors. Originally, the TFEQ was designed to measure the cognitive and behavioral components of eating in obese populations. It contained 51 items aggregated into three scales: cognitive restraint, disinhibition and hunger [15]. The TFEQ was modified by Karlsson et al. [33], who abbreviated it to 18 items (TFEQ-R18) and reconceptualized disinhibition as uncontrolled eating and hunger as emotional eating. The TFEQ-R18 has been found to be valid in the general female sample [13]. The TFEQ-R18 contains three subscales: (1) cognitive restraint (an individual’s tendency to restrict dietary intake to control weight); (2) uncontrolled eating (an individual’s tendency to eat in response to external food cues with episodes of loss of control and overeating); and (3) emotional eating (an individual’s tendency to eat in response to negative emotion). We used the Polish version of the TFEQ-R18 [34]. The reliability estimates for each subscale were excellent: α_Uncontrolled Eating_ = 0.84, α_Emotional Eating_ = 0.86 and α_Cognitive Restraint_ = 0.78 [34]. In the present study, internal reliabilities were as follows: uncontrolled eating Cronbach’s α = 0.871, emotional eating Cronbach’s α = 0.735 and cognitive restraint Cronbach’s α = 0.800. In the present study, we used two subscales of the TFEQ-R18, uncontrolled eating and emotional eating, for assessing disinhibited eating patterns.

#### 2.3.3. The Perseverative Thinking Questionnaire

The Perseverative Thinking Questionnaire (PTQ) [35,36] measures repetitive negative thinking. It contains 15 items in three subscales: (1) the core characteristics of repetitive negative thinking: (a) its repetitive nature, (b) its intrusive nature and (c) the difficulty in disengaging; (2) the perceived unproductiveness of repetitive negative thinking; and (3) capturing mental resources. We used the Polish version of the PTQ [36]. The internal consistency of the PTQ was low but adequate (α = 0.64–0.92) [36]. In the present study, internal reliabilities were as follows: the core characteristics of repetitive negative thinking Cronbach’s α = 0.943, unproductiveness of repetitive negative thinking Cronbach’s α = 0.835 and capturing mental resources Cronbach’s α = 0.855. In the present study, we used three subscales of the PTQ for assessing repetitive negative thinking (rumination): the core characteristics of repetitive negative thinking, the unproductiveness of repetitive negative thinking and capturing mental resources.

## 3. Results

### 3.1. Statistical Analysis

**Figure 1:**
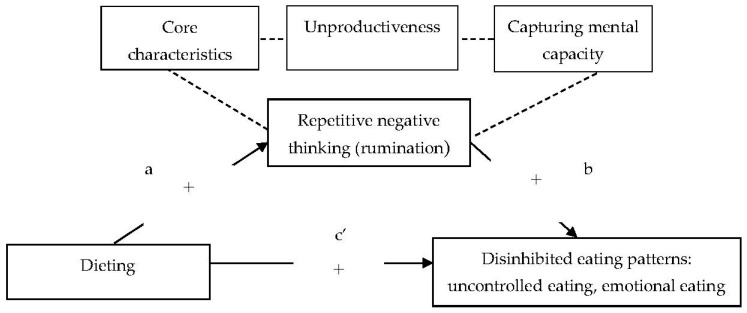
Hypothesized mediation model for relationship between dieting, rumination and both emotional eating and uncontrolled eating in women with normal body weight. Note: + positive prediction.

Statistical analyses were performed using IBM SPSS Statistics for Windows, Version 26.0 (IBM Corp., Armonk, NY, USA). We used the PROCESS version 3.5 macro by Hayes [37] to test the hypothesized mediation models between dieting, rumination and both emotional eating and uncontrolled eating. The results were reported as indirect and direct effects of measured variables. The indirect effect represents the relationship between dieting and both emotional and uncontrolled eating through rumination. A simple pathway analysis with 10,000 bootstrapping samples was conducted. To determine the significance of the mean indirect effects, 95% confidence intervals (CIs) were obtained. If the CI did not contain zero, the indirect effect was considered statistically significant at the 0.05 level. The direct effect represents the relationship between dieting and both emotional eating and uncontrolled eating before adjustment for rumination. The regression analyses were performed for each path, regressing the predictor (dieting; one subscale of the EAT-26) on both the outcome (emotional eating and uncontrolled eating; two subscales of the TFEQ-R18) and the mediator (repetitive negative thinking; three subscales of the PTQ). We estimated the direct effects of the predictor on the mediator (path *a*), the mediator on the outcome (path *b*) and the predictor on the outcome (path *c’*), and the indirect effect (mediation) of the predictor on the outcome via the mediator (path *a* × *b*) (Figure 1). Separate pathway analyses were conducted for each of the three subscales of the PTQ.

### 3.2. Correlations between Study Variables

Prior to the mediational analyses, Pearson correlation coefficients were calculated to examine associations of study variables (Table 1).

### 3.3. Mediation Analysis for Relationship between Dieting, Rumination and Disinhibited Eating Patterns in Women with Normal Body Weight

In order to assess whether the relationship between dieting and disinhibited eating patterns is mediated by rumination, mediation analyses were used (Table 2). The results indicated that for:

1. Core characteristics of repetitive negative thinking: The path *a* model was significant, showing that greater dieting significantly predicted higher levels of core characteristics of repetitive negative thinking (*β* = 0.21, 95% CI [0.04, 0.37]). Path *b* was also significant, indicating that higher levels of core characteristics of repetitive negative thinking significantly predicted more frequent uncontrolled eating (*β* = 0.46, 95% CI [0.20, 0.72]) and emotional eating (*β* = 0.24, 95% CI [0.12, 0.32]). Path *c* was also significant, demonstrating that greater dieting significantly predicted more frequent uncontrolled eating (*β* = 0.83, 95% CI [0.54, 1.13]) and emotional eating (*β* = 0.31, 95% CI [0.16, 0.45]) when core characteristics of repetitive negative thinking were not included in the model.

2. Unproductiveness of repetitive negative thinking: The path *a* model was significant, showing that greater dieting significantly predicted higher levels of unproductiveness of repetitive negative thinking (*β* = 0.25, 95% CI [0.08, 0.42]). Path *b* was also significant, indicating that higher levels of unproductiveness of repetitive negative thinking significantly predicted more frequent uncontrolled eating (*β* = 0.47, 95% CI [0.23, 0.72]) and emotional eating (*β* = 0.25, 95% CI [0.12, 0.37]). Path *c* was also significant, demonstrating that greater dieting significantly predicted more frequent uncontrolled eating (*β* = 0.81, 95% CI [0.52, 1.10]) and emotional eating (*β* = 0.30, 95% CI [0.15, 0.44]) when unproductiveness of repetitive negative thinking was not included in the model.

3. Capturing mental capacity: The path *a* model was significant, showing that greater dieting significantly predicted higher levels of capturing mental capacity (*β* = 0.92, 95% CI [0.45, 1.38]). Path *b* was also significant, indicating that higher levels of capturing mental capacity significantly predicted more frequent uncontrolled eating (*β* = 0.15, 95% CI [0.06, 0.24]) and emotional eating (*β* = 0.09, 95% CI [0.05, 0.13]). Path *c* was also significant, demonstrating that greater dieting significantly predicted more frequent uncontrolled eating (*β* = 0.79, 95% CI [0.49, 1.09]) and emotional eating (*β* = 0.27, 95% CI [0.13, 0.42]) when capturing mental capacity was not included in the model.

## 4. Discussion

Consistent with our first hypothesis, we found that a high level of concern about dieting was related to more frequent uncontrolled eating and emotional eating, and higher levels of core characteristics of repetitive negative thinking, unproductiveness of repetitive negative thinking and capturing mental capacity in a community sample of adult women. Previous studies found that an increased conscious control of eating behavior (i.e., dietary restraint) was associated with obsessive thoughts about forbidden foods [38] and disinhibition [39]. Emotional eating, as a ‘disinhibitor’, requires prior inhibition (i.e., restraint) by definition [40]. Therefore, it can be supposed that individuals with disinhibited eating might exert more dietary restraint at times in order to compensate for their greater disinhibition [41]. The fact that restrained eating is a cause of emotional eating has still not been resolved [42]. Some researchers [43] argue that the impact of overeating is limited by dietary restraint. We could suppose that adult women who diet may engage in emotional eating to cope with negative emotions and thoughts, which in the long term is a maladaptive emotion regulation strategy [44]. On the other hand, increased uncontrolled and emotional eating may be a consequence of women’s dietary restriction, whereas the abstinence from food (dieting) may be the means of regulating the negative effect. This should be explored in future studies.

It is worth pointing out that although there is a strong relationship between uncontrolled eating and emotional eating and they can be conceptualized as different forms of disinhibited eating patterns, it has been empirically shown [45] that they refer to independent constructs. An essential difference between uncontrolled eating and emotional eating is that uncontrolled eating is attributed to a heightened sensitivity to external food cues, regardless of the internal state of hunger and satiety (externality theory), while emotional eating is attributed to a confusion of physiological states accompanying negative emotions with physiological correlates of hunger and satiety (psychosomatic theory) [45]. However, despite their differences, in both uncontrolled eating and emotional eating, individuals’ misperception of their internal state prior to eating is considered to be a causal factor in overeating [45].

Consistent with our second hypothesis, the results showed that repetitive negative thinking (core characteristics of repetitive negative thinking, unproductiveness of repetitive negative thinking and capturing mental capacity) mediated the positive relationships between dieting and both uncontrolled eating and emotional eating in normal-weight women. Thus, rumination could be a cognitive mechanism between dieting and disinhibited eating patterns in these women. Cognitive distortions relevant to food intake may be implicated in difficulties with normalized eating [26]. It could be possible that beliefs about rumination act as an internal control mechanism that strengthens ruminative thinking [46], which may be one factor accounting for its persistence in normal-weight women. This hypothesis needs to be tested in future studies.

Our study may indicate that women with normal body weight do not suppress their negative repetitive thoughts, and that this consequently leads to disinhibited eating patterns. Rumination leads to unhealthy eating behavior [47]. Perhaps, the more time women spend dieting, the more likely they are to avoid food-related thoughts over time (an additive effect of food thought suppression) [48].

Our results are in line with the restraint theory [4], showing that both uncontrolled eating and emotional eating are caused by dieting, rather than preceding dieting (this is in contrast with the psychosomatic theory and externality theory).

In future research, it would be interesting to explore the impact of trying to suppress eating-related thoughts on subsequent eating-related thoughts in women with normal body weight. Findings have shown that individuals practicing dietary restraint and presenting disinhibited eating patterns used thought suppression (the conscious attempt to not think about something) [49]. Moreover, individuals practicing dietary restraint who tend to overeat try to suppress thoughts about food more often, but if they do, they think about food more often afterwards (a rebound effect following a thought suppression task about food). Some studies [50] have shown that food thought suppression increased food-related thoughts, notwithstanding weight status [50], and increased food intake among undergraduate women [51]. Future studies should take the rebound effect following thought suppression for both subsequent thoughts and eating behavior in normal-weight women into consideration. In addition, future studies should include other potential mediators (e.g., weight regulation, weight change, food choices) of relationships between dieting and disinhibited eating patterns.

In conclusion, increasing our understanding of the link between dieting and rumination could potentially inform future prevention programs intended to reduce disinhibited eating patterns in women with normal body weight. In addition, psychological intervention should match with individuals’ eating patterns. Furthermore, the type of disinhibited eating patterns (“habitual” disinhibition—the susceptibility to overeat in response to daily life circumstances, “emotional” disinhibition—the tendency to overeat in response to emotional states and “situational” disinhibition—the susceptibility to overeat in response to specific environmental cues [8]) would be worth investigating further to plan for future intervention.

Some limitations of the current study should be noted. Firstly, the cross-sectional nature of the study means that causal inferences cannot be made. To better examine causality, longitudinal or experimental designs should be used. Therefore, future studies should utilize longitudinal or laboratory-based paradigms to further examine the associations among dieting, rumination and maladaptive eating behaviors. Secondly, the widely used TFEQ-R18 also has restrictions; for example, the use of three items to measure emotional eating might not be sufficiently reliable or valid to reflect emotional eating. In a future study, other measures of emotional eating (e.g., the Emotional Eating Scale [52], the Dutch Eating Behavior Questionnaire [53]) should be used to confirm the findings of the current study. Finally, self-report measures may be affected by the common methods bias.

## 5. Conclusions

Our findings showed that dieting and rumination (core characteristics of repetitive negative thinking, unproductiveness of repetitive negative thinking and capturing mental capacity) were positively associated with each other and both predicted higher levels of uncontrolled eating and emotional eating. The effect of dieting on both uncontrolled eating and emotional eating was mediated by repetitive negative thinking (its core characteristics, its unproductiveness and capturing mental capacity). Some research has explored the impact of trying to suppress eating-related thoughts on subsequent thoughts about eating. Our results suggest that rumination should be taken into consideration in psychological intervention for decreasing disinhibited eating patterns in women with normal body weight.

## Figures and Tables

**Table 1:** Correlations of study variables.

Variable	1	2	3	4	5	6
1. Core characteristics of RNT	-					
2. Unproductiveness of RNT	0.75 ***	-				
3. Capturing mental capacity	0.79 ***	0.84 ***	-			
4. Dieting	0.18 *	0.21 **	0.27 ***	-		
5. Uncontrolled eating	0.30 ***	0.32 ***	0.32 ***	0.41 ***	-	
6. Emotional eating	0.31 ***	0.33 ***	0.37 ***	0.33 ***	0.61 ***	-

Note: * *p* < 0.05; ** *p* < 0.01; *** *p* < 0.005; RNT: repetitive negative thinking.

**Table 2:** Mediation analysis for relationship between dieting, rumination and disinhibited eating patterns in women with normal body weight.

	Uncontrolled Eating		Emotional Eating	
	Direct Effect	Indirect Effect	Direct Effect	Indirect Effect
Core characteristics of RNT	Effect = 0.83	Effect = 0.09	Effect = 0.31	Effect = 0.05
	SE = 0.14	BootSE = 0.05	SE = 0.07	BootSE = 0.02
	95% CI [0.54, 1.13]	95% CI [0.01, 0.21]	95% CI [0.16, 0.45]	95% CI [0.00, 0.11]
Unproductiveness of RNT	Effect = 0.81	Effect = 0.12	Effect = 0.30	Effect = 0.06
	SE= 0.14	BootSE = 0.05	SE = 0.07	BootSE = 0.02
	95% CI [0.52, 1.10]	95% CI [0.02, 0.24]	95% CI [0.15, 0.44]	95% CI [0.01, 0.12]
Capturing mental capacity	Effect = 0.79	Effect = 0.02	Effect = 0.27	Effect = 0.08
	SE = 0.15	BootSE = 0.02	SE = 0.07	BootSE = 0.02
	95% CI [0.49, 1.09]	95% CI [0.05, 0.26]	95% CI [0.13, 0.42]	95% CI [0.03, 0.15]

Note: RNT: repetitive negative thinking; SE: standard error; CI: confidence interval.

## Data Availability

The dataset used during the current study are available from the corresponding author on reasonable request.
